# Intraoperative and postoperative feasibility and safety of total tubeless, tubeless, small-bore tube, and standard percutaneous nephrolithotomy: a systematic review and network meta-analysis of 16 randomized controlled trials

**DOI:** 10.1186/s12894-017-0239-x

**Published:** 2017-06-27

**Authors:** Joo Yong Lee, Seong Uk Jeh, Man Deuk Kim, Dong Hyuk Kang, Jong Kyou Kwon, Won Sik Ham, Young Deuk Choi, Kang Su Cho

**Affiliations:** 1 Department of Urology, Severance Hospital, Urological Science Institute, Yonsei University College of Medicine, Seoul, South Korea; 2 Department of Urology, Gyeongsang National University Hospital, Gyeongsang National University School of Medicine, Jinju, South Korea; 3 Department of Radiology, Severance Hospital, Research Institute of Radiological Science, Yonsei University College of Medicine, Seoul, South Korea; 4 Department of Urology, Inha University School of Medicine, Incheon, South Korea; 5 Department of Urology, Severance Check-Up, Yonsei University Health System, Seoul, South Korea; 6 Department of Urology, Gangnam Severance Hospital, Urological Science Institute, Yonsei University College of Medicine, 211 Eonju-ro, Gangnam-gu, Seoul, 06273 South Korea

**Keywords:** Calculi, Lithotripsy, Nephrostomy, Percutaneous, Meta-analysis, Bayes theorem

## Abstract

**Background:**

Percutaneous nephrolithotomy (PCNL) is performed to treat relatively large renal stones. Recent publications indicate that tubeless and total tubeless (stentless) PCNL is safe in selected patients. We performed a systematic review and network meta-analysis to evaluate the feasibility and safety of different PCNL procedures, including total tubeless, tubeless with stent, small-bore tube, and large-bore tube PCNLs.

**Methods:**

PubMed, Cochrane Central Register of Controlled Trials, and EMBASE™ databases were searched to identify randomized controlled trials published before December 30, 2013. One researcher examined all titles and abstracts found by the searches. Two investigators independently evaluated the full-text articles to determine whether those met the inclusion criteria. Qualities of included studies were rated with Cochrane’s risk-of-bias assessment tool.

**Results:**

Sixteen studies were included in the final syntheses including pairwise and network meta-analyses. Operation time, pain scores, and transfusion rates were not significantly different between PCNL procedures. Network meta-analyses demonstrated that for hemoglobin changes, total tubeless PCNL may be superior to standard PCNL (mean difference [MD] 0.65, 95% CI 0.14–1.13) and tubeless PCNLs with stent (MD -1.14, 95% CI -1.65–-0.62), and small-bore PCNL may be superior to tubeless PCNL with stent (MD 1.30, 95% CI 0.27–2.26). Network meta-analyses also showed that for length of hospital stay, total tubeless (MD 1.33, 95% CI 0.23–2.43) and tubeless PCNLs with stent (MD 0.99, 95% CI 0.19–1.79) may be superior to standard PCNL. In rank probability tests, small-bore tube and total tubeless PCNLs were superior for operation time, pain scores, and hemoglobin changes.

**Conclusions:**

For hemoglobin changes, total tubeless and small-bore PCNLs may be superior to other methods. For hospital stay, total tubeless and tubeless PCNLs with stent may be superior to other procedures.

## Background

Urinary stone is one of the most prevalent urological disorders. Reports suggest that up to 12% of people will suffer from urinary tract calculi during their lifetime, and the rates of recurrence is close to 50% [1]. There are several treatment modalities for renal stones, including observation expecting spontaneous passage, extracorporeal shock wave lithotripsy (ESWL), percutaneous nephrolithotomy (PCNL), and retrograde intrarenal surgery (RIRS) using flexible ureterorenoscope [2]. PCNL is currently the standard treatment for large renal stones considered too large for or refractory to shock wave lithotripsy [3, 4]. Conventionally, a 20-24 French nephrostomy catheter is placed routinely after PCNL to provide urine drainage, prevent extravasation of urine, and make tamponade against bleeding [5, 6]. In addition, it can be used as a tract for a second-look PCNL [7]. The need for placing a conventional large-bore nephrostomy catheter has been questioned because of its accompanying increase in postoperative discomfort and other morbidity, and the low incidence of second-look operations [8, 9]. In recent years, tubeless or small-bore PCNL has been widely used, and previously reported systematic reviews have demonstrated the safety and efficacy in these techniques.

The recently introduced network meta-analysis is a meta-analysis in which multiple treatments are compared using both direct comparisons of interventions within randomized controlled trials (RCTs), and indirect comparisons across trials based on a common comparator [10–14]. Thus, we performed a systematic review and network meta-analysis based on published relevant studies to evaluate the feasibility and safety of each PCNL procedure, including total tubeless, tubeless with stent, small-bore tube, and large-bore tube PCNLs, for the treatment of renal stones.

## Methods

### Inclusion and exclusion criteria

Reported RCTs that fitted the following criteria were selected: (i) a design of each study that involved comparing the feasibility and safety for least two PCNL procedures, including total tubeless, tubeless with stent, small-bore tube, and large-bore tube PCNLs; (ii) the study groups were matched for baseline characteristics, including the total number of subjects and the values of each variable; (iii) at least one of the following outcomes was assessed: operation time, hospital stay length, hemoglobin decrease, return to normal activity, and complication rate; and (iv) the full text of each study was accessible and written in English.

The exclusion criteria were as follows: (i) noncomparative studies; (ii) the trial included children; and (iii) the trial did not exclude patients who underwent bilateral simultaneous PCNL or had complete or partial staghorn stones, more than two nephrostomy tracts, anatomical anomalies, or urinary infection. This report was prepared in compliance with the Preferred Reporting Items for Systematic Reviews and Meta-Analyses (PRISMA) statement (accessible at http://www.prisma-statement.org/) [15].

### Search strategy

A literature search was performed to identify RCTS published prior to December 30, 2013 in PubMed, the Cochrane Central Register of Controlled Trials, and EMBASE™ online databases. A cross-reference search of eligible articles was performed to identify additional studies not found by the computerized search. Combinations of the following MeSH and key words were used: percutaneous nephrolithotomy or nephrostomy or percutaneous nephrostomy or nephrolithiasis or PCNL or PCN or PNL, and total tubeless or tubeless or nephrostomy free.

### Data extraction

One researcher (J.Y.L.) screened the title and abstract of all articles retrieved using the search strategy. The other two investigators (D.H.K. and H.L.) independently assessed the full text of the articles to determine whether they met the inclusion criteria. For each included study, the following data were extracted independently as follows; authors, date, demographics of included patients, PCNL methods, feasibility, efficacy outcomes, complications, and inclusion of a reference standard. Disagreements arising in the study selection and data extraction processes were resolved by discussion until a consensus was reached or by arbitration employing another researcher (K.S.C.).

### Study quality assessment

Once the final group of articles was agreed upon, two researchers (J.Y.L. and D.H.K.) independently examined the quality of each article using the Cochrane’s risk-of-bias as a quality assessment tool for RCTs. The assessment involves the assignment of a “yes,” “no,” or “unclear” rating for each domain, designating a low, high, or unclear risk of bias, respectively. If ≤1 domain was rated “unclear” or “no,” the study was classified as having a low risk of bias. If ≥4 domains were rated “unclear” or “no,” the study was classified as having a high risk of bias. If 2 or 3 domains were rated “unclear” or “no,” the study was classified as having a moderate risk of bias. [16]. Quality assessment was performed using Review Manager 5.2 (RevMan 5.2.11, Cochrane Collaboration, Oxford, UK).

### Statistical analyses

Each outcome variable at specific time-points was compared by network meta-analysis using the odds ratio (OR) or mean difference (MD) with 95% confidence interval (CI). A random-effect model was used. Each analysis was based on non-informative priors for effect size and precision. Convergence and lack of auto-correlation were checked and confirmed after four chains and a 50,000-simulation burn-in phase, and direct probability statements were based on an additional 100,000-simulation phase. Calculation of the probability that each group had the lowest rate of clinical events was performed using Bayesian Markov Chain Monte Carlo modeling. Sensitivity analyses were performed by repeating the main computations using a fixed-effect method. Model fit was appraised by computing and comparing estimates for deviance and deviance information criterion. Pairwise inconsistency and inconsistency between direct and indirect effect estimates were assessed with the I^2^-statistic, with values <25%, 25% to 50%, and >50% representing mild, moderate, and severe inconsistency, respectively. The extent of small study effects/publication bias was assessed by visual inspection of funnel plots for the pairwise meta-analyses. All statistical analyses were performed using Review Manager 5 and R (R version 3.0.3, R Foundation for Statistical Computing, Vienna, Austria; http://www.r-project.org) [17], and its meta, forestplot, gemtc, and R2WinBUGS packages for pairwise and network meta-analyses using Bayesian Markov Chain Monte Carlo modeling.

## Results

### Eligible studies

**Fig. 1:**
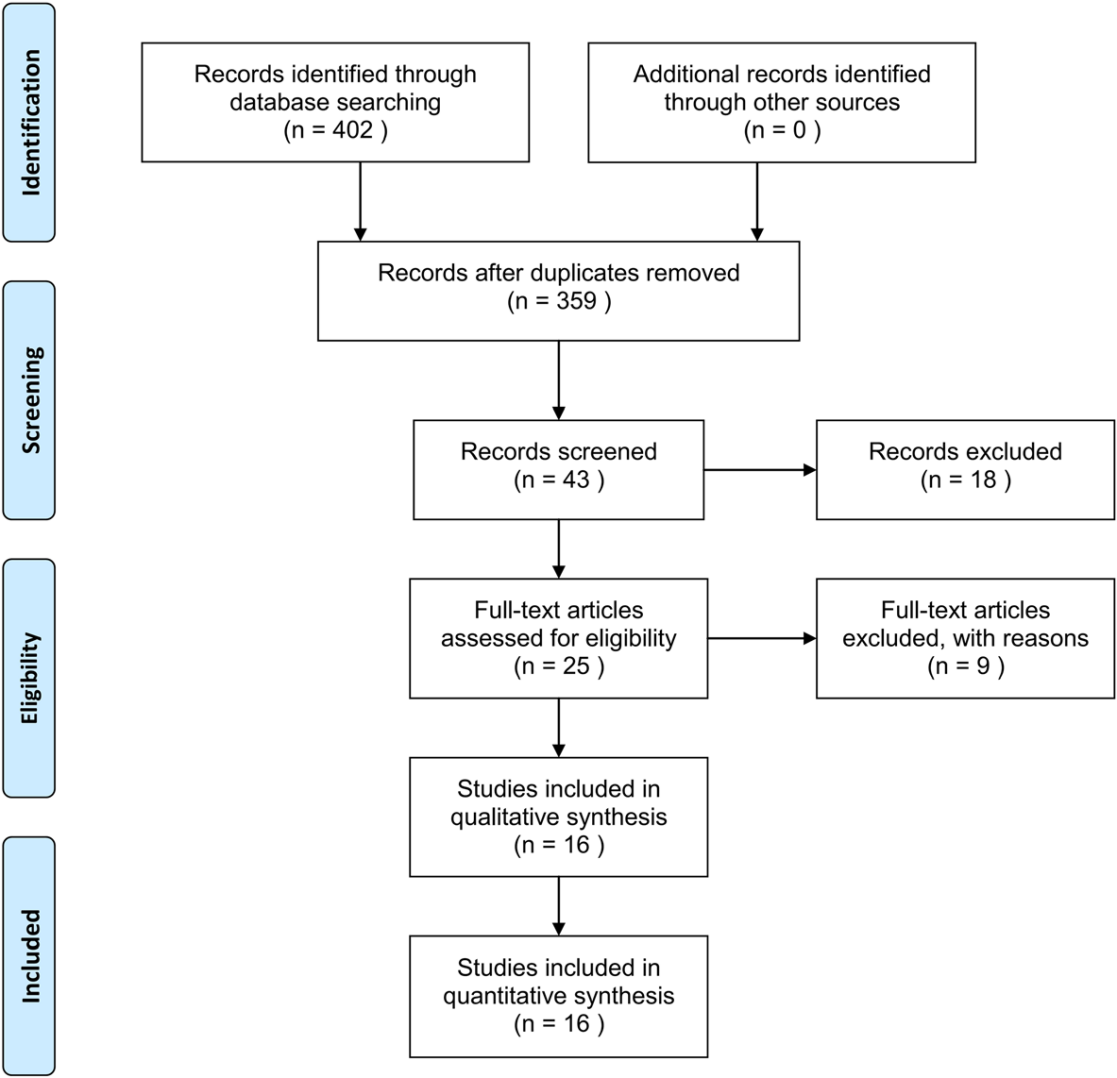
Search strategy for a systematic review and meta-analysis to compare the feasibility and safety of different PCNL procedures, including total tubeless, tubeless with stent, small-bore tube, and large-bore tube PCNLs

Our database search identified 43 studies that could be potentially included in the meta-analysis. Based on the inclusion and exclusion criteria, 18 articles were excluded during screening of the titles and abstracts because they were retrospective studies (11 articles) or case series (7 articles). This left 25 RCTS that evaluated various types of PCNL procedures for renal stones. After reviewing the full-text articles for these studies, 9 were excluded because they reported irrelevant results. Therefore, 16 RCTs were ultimately included in the qualitative analysis, as well as the quantitative synthesis using pairwise and network meta-analyses (Fig. 1).

**Fig. 2:**
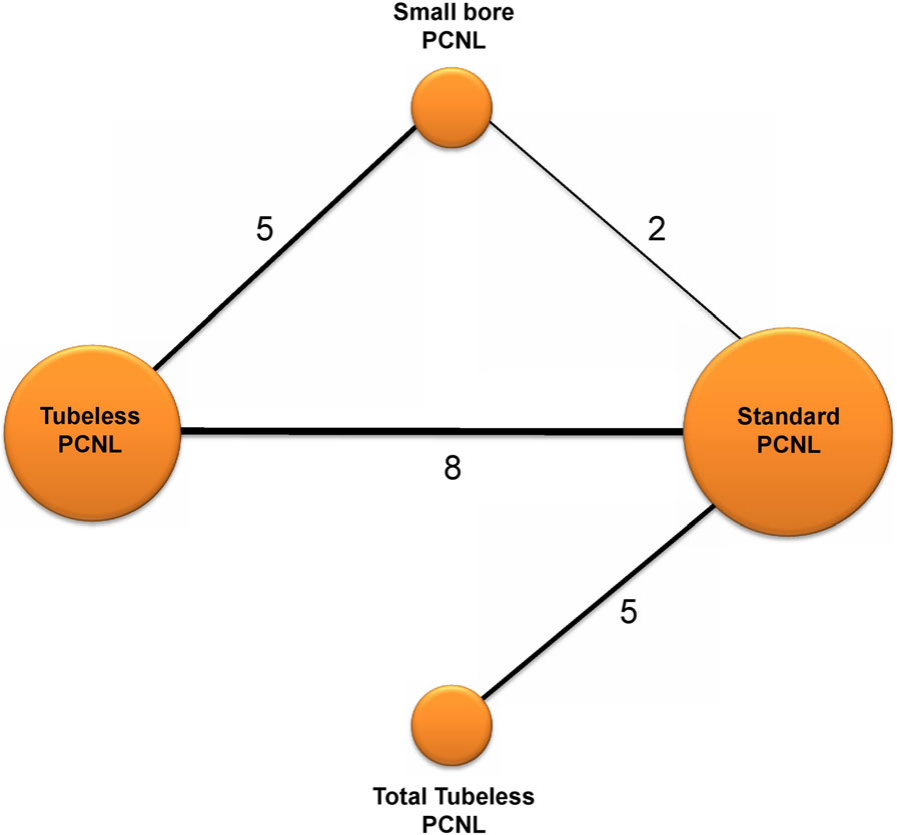
Comparison network of included randomized controlled trials. Five studies included comparisons between standard and total tubeless PCNLs, and five RCTs also compared standard and tubeless PCNLs. Four trials reported on various factors in small-bore and tubeless PCNLs. In two studies, the results of three arms—standard, small-bore, and tubeless PCNLs—were published

There were differences in procedures among the included studies. Five studies included comparisons between standard and total tubeless PCNLs, and five RCTs also compared standard and tubeless PCNLs. Four trials reported on various factors in small-bore and tubeless PCNLs. In two studies, the results of three arms—standard, small-bore, and tubeless PCNLs—were published (Table 1). Finally, the included studies covered four different PCNL procedures: total tubeless, tubeless, standard and small-bore PCNLs (Fig. 2).

**Table 1:** Characteristics of included trials

Study	Year	Design	Procedures	Sample size	Age (year)	Stone burden		Tube	Stone-free rate		Quality assessment
						Size	*P*-value		(%)	*P*-value	
Chang et al. [41].	2011	RCT	Standard	63	58.7	24.86 ± 2.78 mm	0.722	20 Fr (7 Fr)	75%	0.51	Low
			Total tubeless	68	59.2	24.74 ± 2.69 mm		None	74%		
Aghamir et al. [42].	2011	RCT	Standard	35	40	2.87 ± 0.62 cm^2^	0.66	NA	83%	NA	Low
			Total tubeless	35	38.4	2.81 ± 0.59 cm^2^		None	86%		
Kara et al. [43].	2010	RCT	Standard	30	66.5	25.6 mm	NA	18 Fr	90%	>0.05	Low
			Total tubeless	30	67.7	22.3 mm		None	96%		
Mishra et al. [44].	2010	RCT	Standard	11	42.5	2737 μL	0.18	20 Fr	81.8%	0.14	Low
			Tubeless	11	42.3	2934.2 μL		None (6 Fr)	72.7%		
Istanbulluoglu et al. [45].	2009	RCT	Standard	45	43.9	432.35 ± 195.97 mm^2^	0.46	14 Fr	NA	NA	Low
			Total tubeless	45	47.5	448.93 ± 249.13 mm^2^		None	NA		
Crook et al. [46].	2008	RCT	Standard	25	53	17.5 mm	NA	26 Fr	84%	NA	Low
			Total tubeless	25	52	21.6 mm		None	96%		
Agrawal et al. [47].	2008	RCT	Standard	101	31	NA	NA	16 Fr	100%	–	Low
			Tubeless	101	33	NA		None (6 Fr)	100%		
Singh et al. [48].	2008	RCT	Standard	30	34	800 mm^2^	>0.05	22 Fr	93.3%	0.64	Moderate
			Tubeless	30	31	750 mm^2^		None (NA)	90%		
Shah et al. [18].	2008	RCT	Small-bore tube	32	46.7	495.92 mm^2^	0.88	8Fr	87.5%	0.96	Low
			Tubeless	33	44.1	535.36 mm^2^		None (6 Fr)	87.9%		
Sofikerim et al. [35].	2007	RCT	Standard	24	54.1	425 mm^2^	NA	24 Fr or 18 Fr	85% (24 Fr),	0.71	Moderate
			Tubeless	24	47.8	428 mm^2^		None (6 Fr)	83% (18 Fr), 79%		
Tefekli et al. [49].	2007	RCT	Standard	18	41.32	3.1 cm	NA	14 Fr	89%	>0.05	Moderate
			Tubeless	17	38.4	2.8 cm		None (NA)	94%		
Weiland et al. [50].	2007	RCT	Small-bore tube	9	65	6.7 cm^2^	0.15	8.3 Fr			Moderate
			Tubeless	9	54	3.2 cm^2^		None (8.2 Fr)			
Choi et al. [32].	2006	RCT	Small-bore tube	12	47	32.41 mm	0.77	8.2 Fr	91.7%	0.64	High
			Tubeless	12	52.9	28.5 mm		None (6 Fr)	100%		
Desai et al. [51].	2004	RCT	Standard	10	43.4	263.7 mm^2^	>0.05	20 Fr	100%	–	Moderate
			Small-bore tube	10	44.8	243 mm^2^		9 Fr	100%		
			Tubeless	10	41.1	249.1 mm^2^		None (6 Fr)	100%		
Marcovich et al. [52].	2004	RCT	Standard	20	58	3.6 cm	0.64	24 Fr		0.63	Moderate
			Small-bore tube	20	61	3 cm		8 Fr			
			Tubeless	20	57	3.4 cm		None (NA)			
Feng et al. [53].	2001	RCT	Standard	10	53	8.4 cm^3^	0.75	22 Fr	31.5%	NA	Moderate
			Tubeless	8	62	4.4 cm^3^		None (NA)	71.4%		

*NA* not applicable, *RCT* randomized controlled trial

### Quality assessment and publication bias

**Fig. 3:**
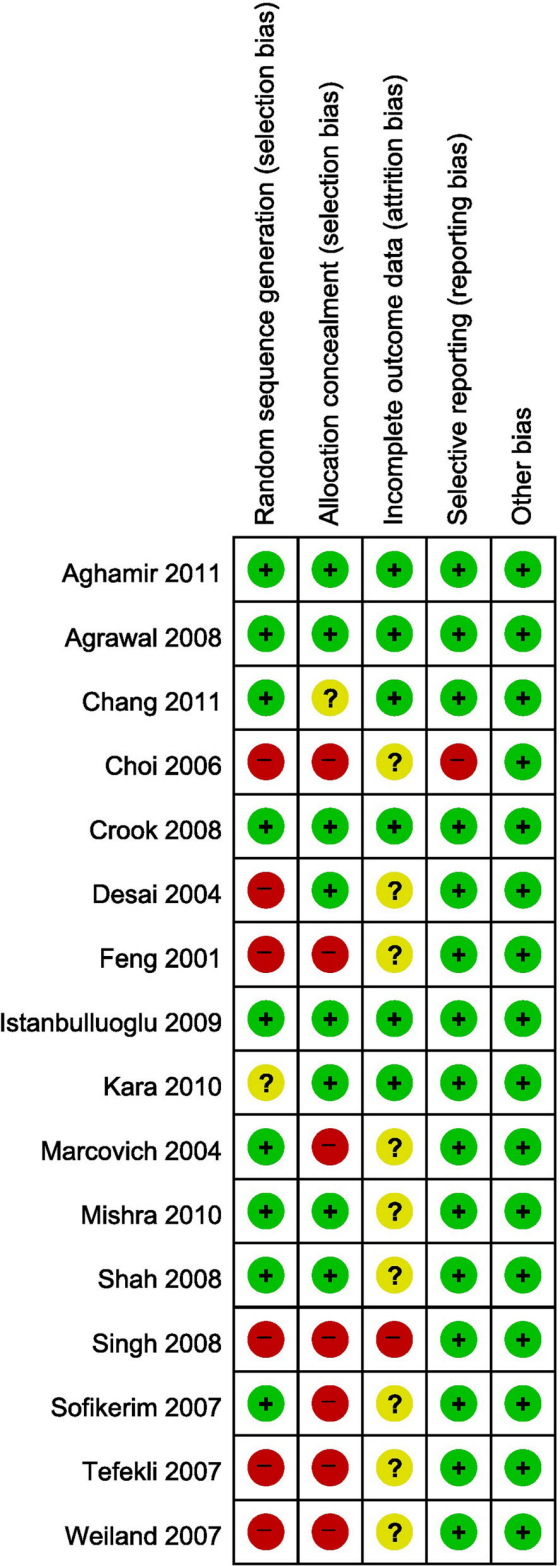
Risk-of-bias summary: review of the authors’ judgments on each risk-of-bias item for each included study

**Fig. 4:**
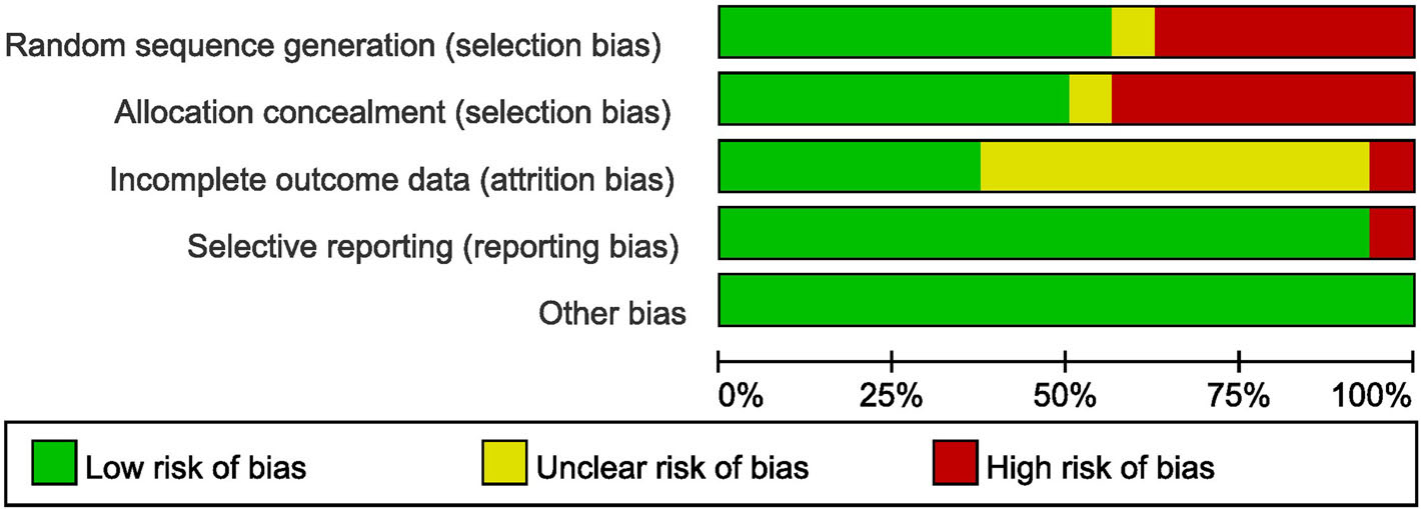
Risk-of-bias graph: review of the authors’ judgments on each risk-of-bias item presented as percentages across all included studies

**Fig. 5:**
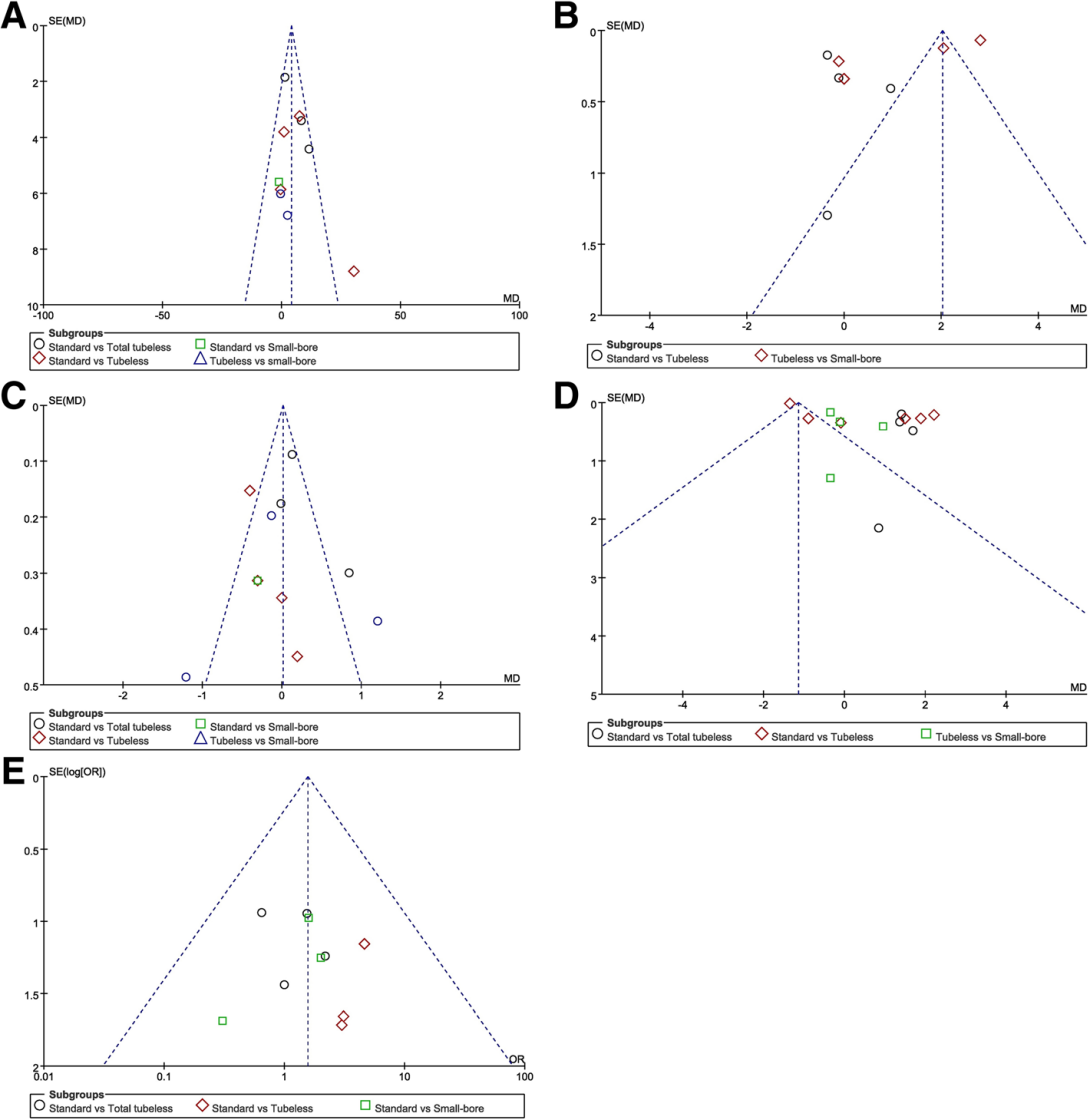
Funnel plots of each variable. **a** Operation time, **b** visual analogue scale (VAS) pain score, **c** hemoglobin change, **d** length of stay, and **e** transfusion rate. For operation time, hemoglobin change, and transfusion rate, little evidence of publication bias was demonstrated on visual or statistical examination of the funnel plots; however, for VAS scores and hospital stay, moderate evidence of publication bias was demonstrated on visual or statistical examination of the plots

Figures 3 and 4 present the details of quality assessment, as measured by the Cochrane Collaboration risk-of-bias tool. Seven trials exhibited a moderate risk of bias for all quality criteria and only one study was classified as having a high risk of bias (Table 1). For operation time, hemoglobin change, and transfusion rate, little evidence of publication bias was demonstrated on funnel plots; however, for the visual analogue scale (VAS) pain score and hospital stay, moderate evidence of publication bias was demonstrated on these plots (Fig. 5).

### Operation time

**Fig. 6:**
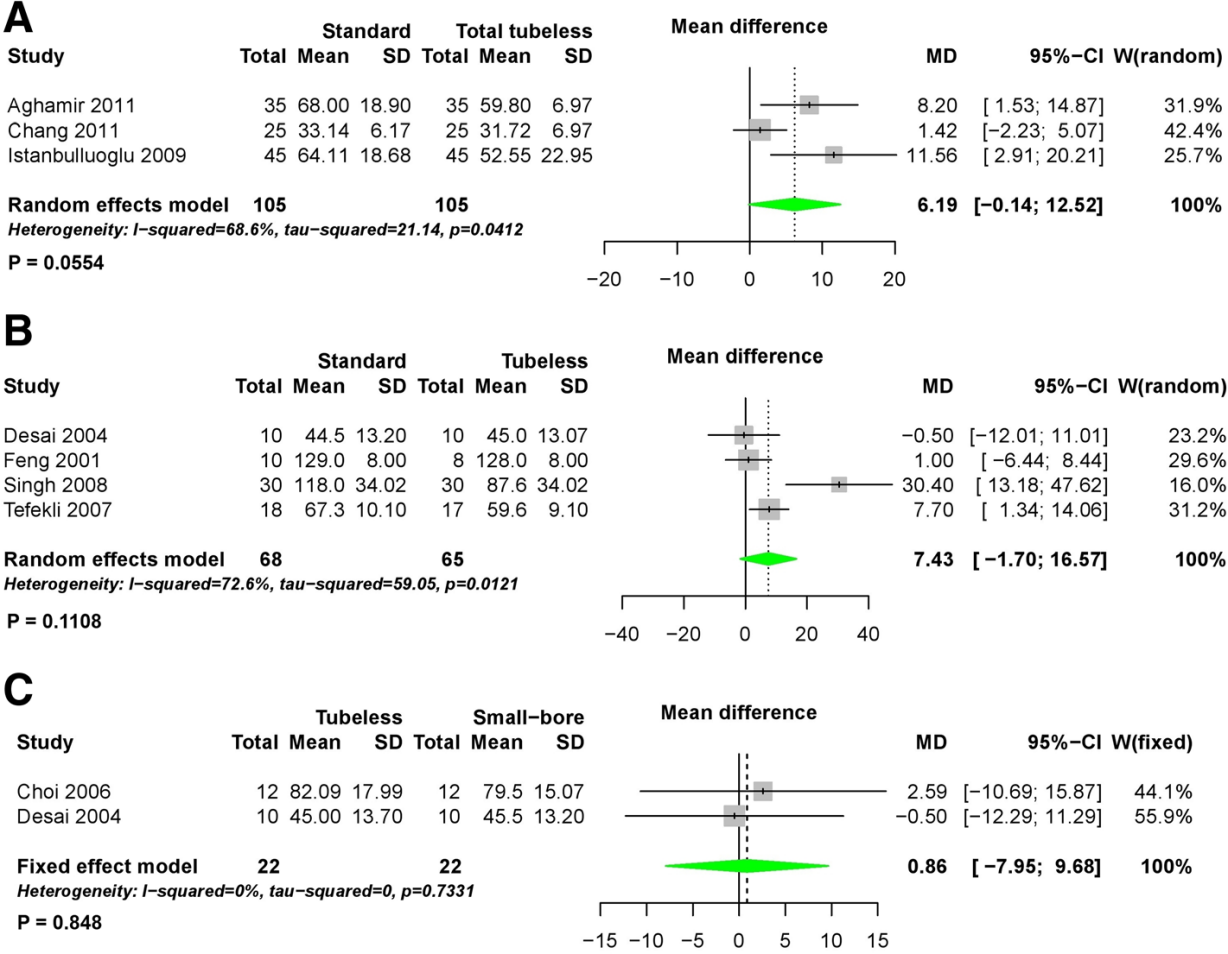
Forest plots for operation time using pairwise meta-analysis. **a** Standard versus total tubeless PCNLs, **b** standard versus tubeless PCNLs, and **c** tubeless versus small-bore PCNLs. SD, standard deviation; MD, Mean difference; CI, confidence interval; W, Weight

**Fig. 7:**
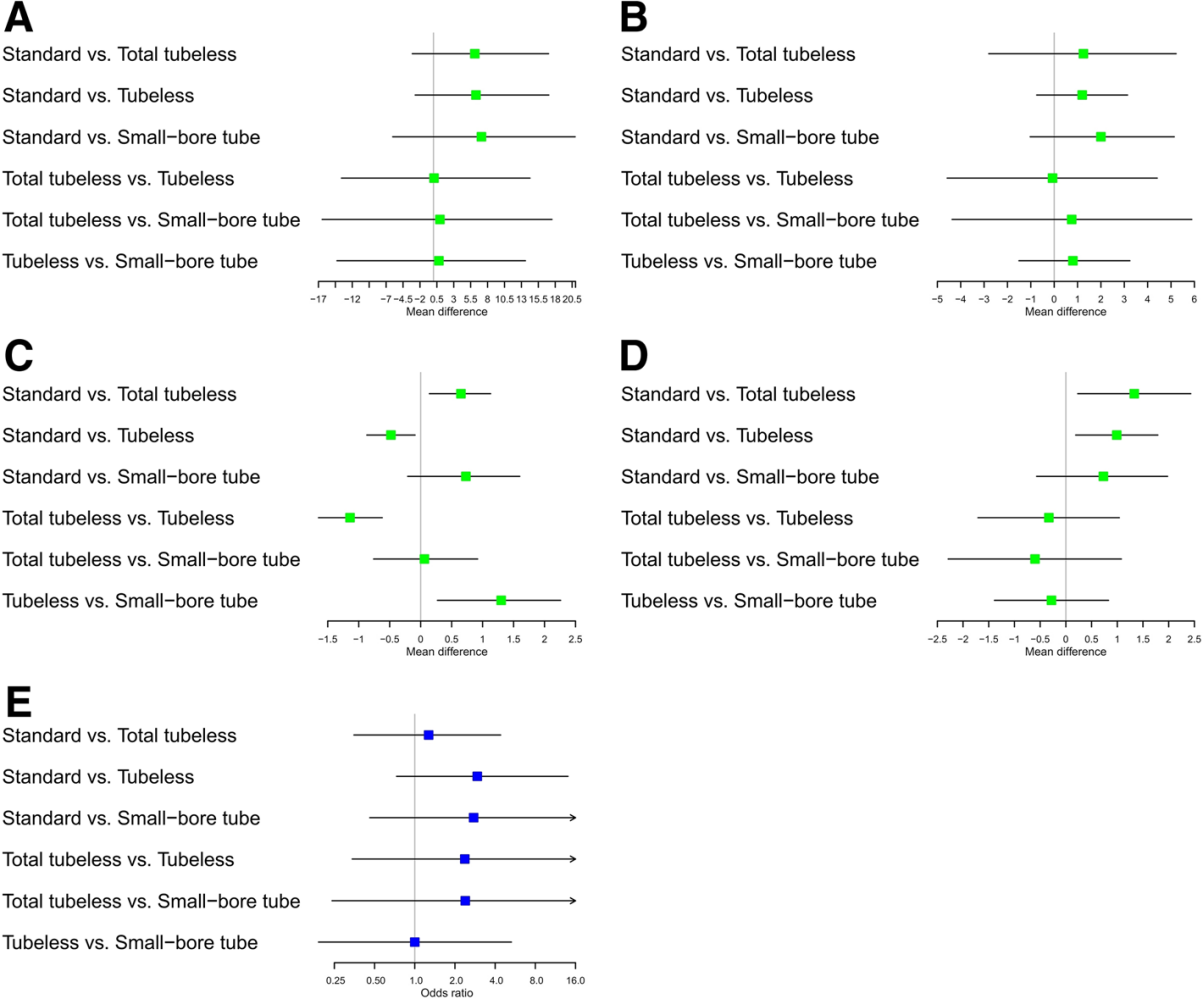
Forest plots for (**a**) operation time, **b** visual analogue scale, **c** hemoglobin change, **d** hospital stay, and **e** transfusion rate using network meta-analysis

**Fig. 8:**
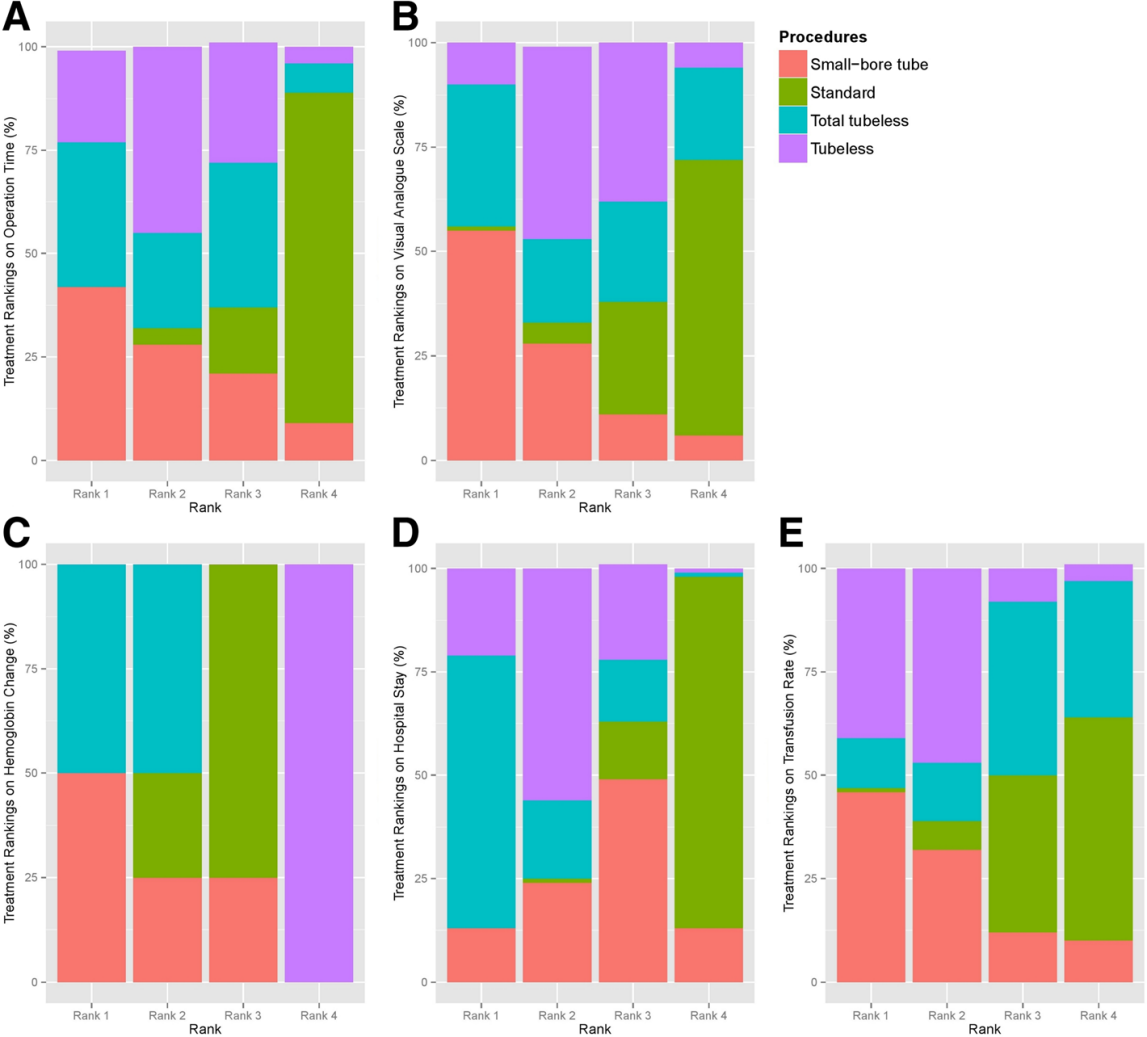
Rank probability test. **a** Operation time, **b** visual analogue scale, **c** hemoglobin change, **d** hospital stay, and **e** transfusion rate

During the pairwise meta-analysis of operation time between standard and total tubeless PCNLs, there was a significant degree of heterogeneity among these studies, and data were pooled with a random effects model (*P* = 0.04, I^2^ = 69%). There was no statistically significant difference in operation time between standard and total tubeless PCNLs, although the MD was 6.19 (95% CI -0.14 to 12.52) (Fig. 6a). Between standard and tubeless PCNLs with stent, the MD also demonstrated no statistical difference (MD 7.43, 95% CI -1.70 to 16.57) (Fig. 6b). Likewise, the MDs did not exhibit statistically significant differences for standard versus small-bore PCNLs (MD -1.0, 95% CI -11.93 to 9.93) or tubeless versus small-bore PCNLs (MD 0.86, 95% CI -7.95 to 9.68) (Fig. 6c). Using network meta-analysis, there were no significant differences among all procedures (Fig. 7a) (Table 2), although total tubeless and small-bore PCNLs had higher rank probabilities than the other procedures (Fig. 8a).

**Table 2:** Results of network and pairwise meta-analyses comparing procedures for operation time, visual analogue scale pain score, hemoglobin change, and hospital stay

Procedures	Network meta-analysis		Pairwise meta-analysis	
	Mean difference	95% CI	Mean difference	95% CI
Operation time				
Standard				
Total tubeless	6.11	−3.14 – 17.02	6.19^a^	−0.14 – 12.52
Tubeless	6.28	−2.71 – 17.06	7.43^a^	−1.70 – 16.57
Small-bore tube	7.09	−6.03 – 20.95	NA	
Total tubeless				
Tubeless	0.08	−13.60 – 14.27	NA	
Small-bore tube	0.95	−16.46 – 17.52	NA	
Tubeless				
Small-bore tube	0.80	−14.27 – 13.60	0.86^b^	−7.95 – 9.68
Visual analogue scale pain score				
Standard				
Total tubeless	1.25	−2.80 – 5.22	NA	
Tubeless	1.20	−0.75 – 3.14	0.06^a^	−0.56 – 0.69
Small-bore tube	2.00	−1.03 – 5.14	NA	
Total tubeless				
Tubeless	−0.07	−4.58 – 4.41	NA	
Small-bore tube	0.75	−4.37 – 5.89	NA	
Tubeless				
Small-bore tube	0.80	−1.51 – 3.24	1.21^a^	−0.02 – 2.44
Hemoglobin change				
Standard				
Total tubeless	0.65	0.14 – 1.13	0.23^a^	−0.12 – 0.58
Tubeless	−0.48	−0.87 – −0.09	-0.29^a^	−0.53 – −0.05
Small-bore tube	0.73	−0.21 – 1.60	NA	
Total tubeless				
Tubeless	−1.14	−1.65 – −0.62	NA	
Small-bore tube	0.06	−0.76 – 0.92	NA	
Tubeless				
Small-bore tube	1.30	0.27 – 2.26	−0.02^a^	−1.13 – 1.10
Hospital stay				
Standard				
Total tubeless	1.33	0.23 – 2.43	1.42^b^	1.10 – 1.75
Tubeless	0.99	0.19 – 1.79	0.54^a^	−1.03 – 2.11
Small-bore tube	0.73	−0.57 – 1.98	NA	
Total tubeless				
Tubeless	−0.33	−1.71 – 1.04	NA	
Small-bore tube	−0.60	−2.29 – 1.08	NA	
Tubeless				
Small-bore tube	−0.28	−1.39 – 0.83	0.06^a^	−0.56 – 0.69

*CI* confidence interval, *NA* not applicable

^a^Random-effect model with inverse variance method

^b^Fixed-effect model with inverse variance method

### Visual analogue scale pain score

**Fig. 9:**
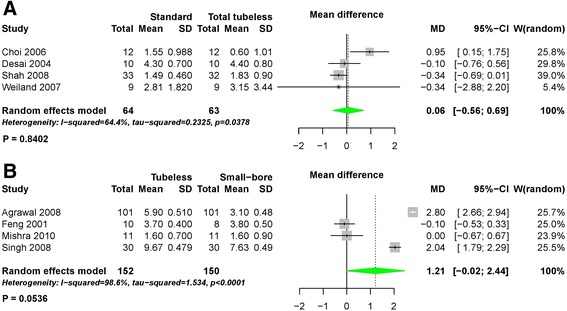
Forest plots for visual analogue scale pain score using pairwise meta-analysis. **a** Standard versus total tubeless PCNLs, and **b** standard versus tubeless PCNLs, SD, standard deviation; MD, Mean difference; CI, confidence interval; W, Weight

In the pairwise meta-analysis of VAS pain scores, there was a significant degree of heterogeneity among studies and the data were pooled with a random effects model. There were no statistically significant differences comparing standard versus total tubeless PCNLs with stent (MD 0.06, 95% CI -0.56 to 0.69, *P* = 0.84) (Fig. 9a) or tubeless versus small-bore PCNLs (MD 1.21, 95% CI -0.02 to 2.44, *P* = 0.05) (Fig. 9b). In the network meta-analysis, there were no statistically significant differences among all procedures for VAS pain scores (Fig. 7b) (Table 2), although the rank probabilities demonstrated that small-bore and total tubeless PCNLs may be superior to the other procedures (Fig. 8b).

### Hemoglobin change

**Fig. 10:**
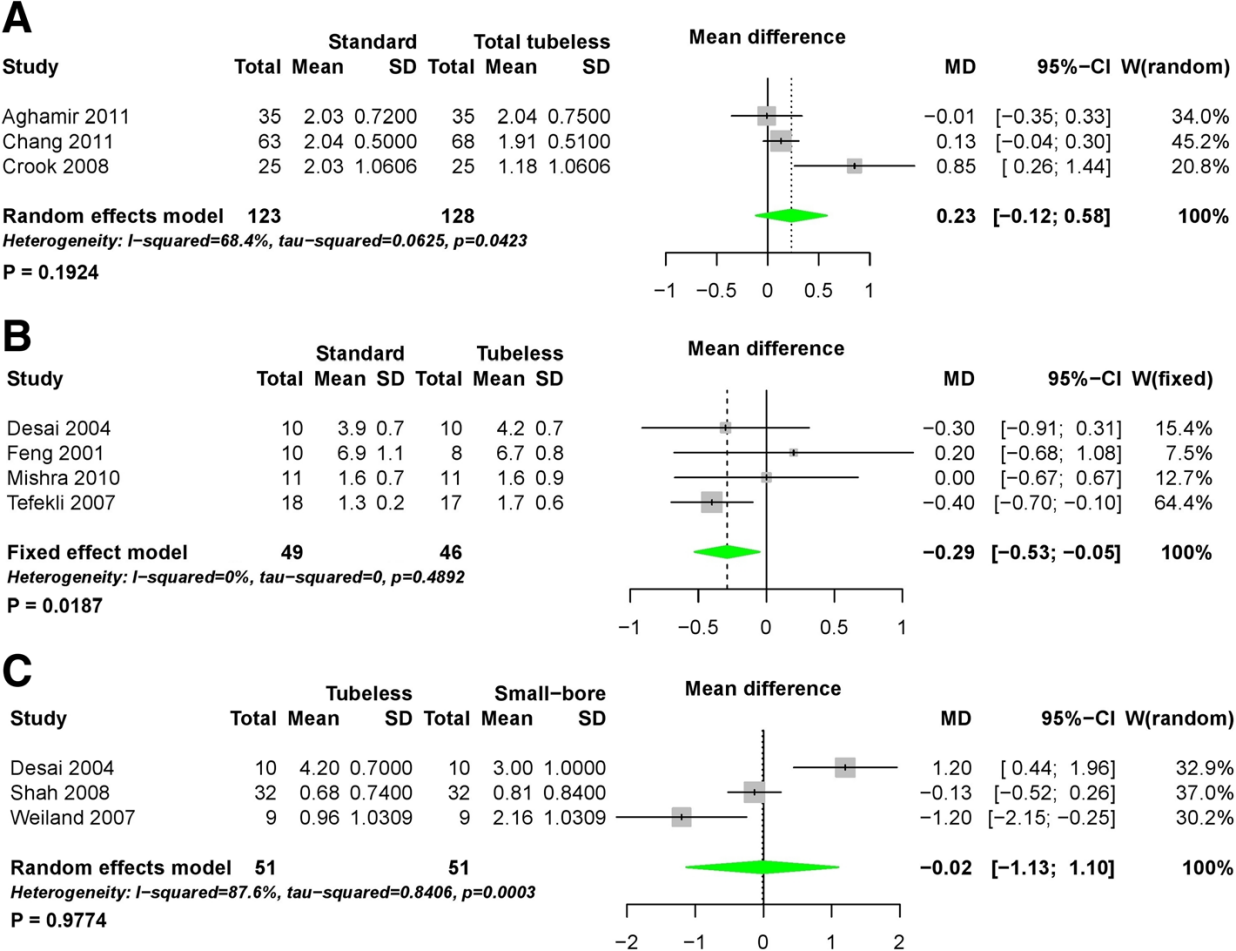
Forest plots for hemoglobin change using pairwise meta-analysis. **a** Standard versus total tubeless PCNLs, **b** standard versus tubeless PCNLs, and **c** tubeless versus small-bore PCNLs. SD, standard deviation; MD, Mean difference; CI, confidence interval; W, Weight

Using pairwise meta-analysis for hemoglobin change, three comparisons, including standard versus total tubeless PCNLs, standard versus tubeless PCNLs with stent, and tubeless versus small-bore PCNLs, were examined (Fig. 10). Only one comparison for standard versus tubeless PCNLs with stent showed a statistically significant difference (MD -0.29, 95% CI -0.53 to −0.05, *P* = 0.02) (Fig. 10b). Network meta-analysis demonstrated that total tubeless PCNL may be superior to standard PCNL (MD 0.65, 95% CI 0.14 to 1.13). Total tubeless (MD -1.14, 95% CI -1.65 to −0.62), and small-bore PCNLs (MD 1.30, 95% CI 0.27 to 2.26) were also superior to tubeless PCNL with stent for hemoglobin change (Fig. 7c) (Table 2). In rank probabilities, total tubeless and small-bore PCNLs were ranked higher than the other procedures (Fig. 8c).

### Hospital stay

**Fig. 11:**
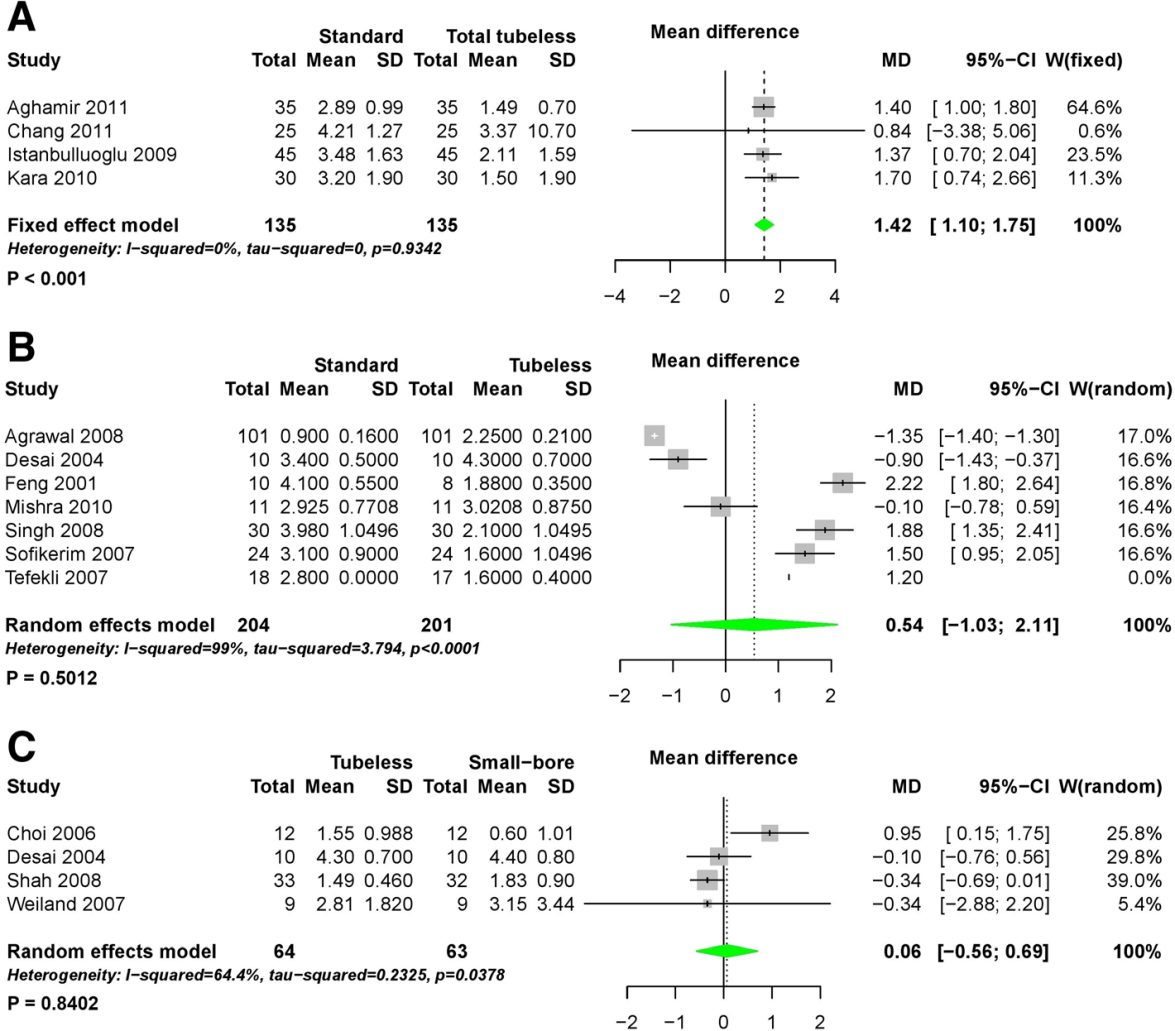
Forest plots for hospital stay using pairwise meta-analysis. **a** Standard versus total tubeless PCNLs, **b** standard versus tubeless PCNLs, and **c** tubeless versus small-bore PCNLs. SD, standard deviation; MD, Mean difference; CI, confidence interval; W, Weight

The length of hospital stay in patients who underwent total tubeless PCNL was shorter than for those who underwent standard PCNL (MD 1.42, 95% CI 1.10 to 1.75, *P* < 0.01) during pairwise meta-analysis (Fig. 11). Network meta-analysis also demonstrated that total tubeless (MD 1.33, 95% CI 0.23 to 2.43) and tubeless PCNLs with stent (MD 0.99, 95% CI 0.19 to 1.79) may be superior to standard PCNL, producing a shorter hospital stay (Fig. 7d). However, there was no significant difference between total tubeless and tubeless PCNLs with stent (MD -0.33, 95% CI -1.71 to 1.04) (Table 2), although total tubeless PCNL showed the highest rank probability of all procedures (Fig. 8d).

### Transfusion rate

**Fig. 12:**
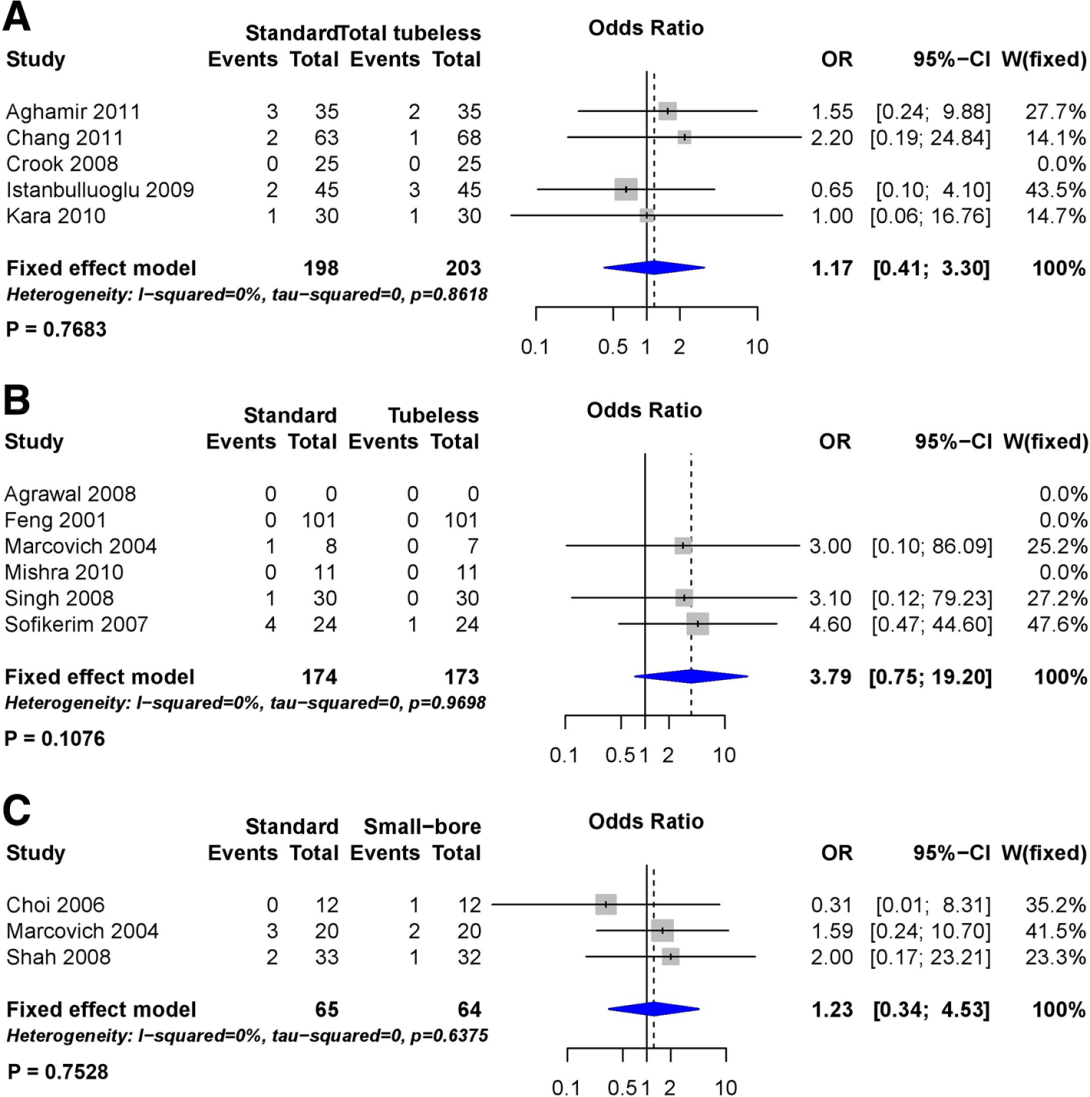
Forest plots for transfusion rate using pairwise meta-analysis. **a** Standard versus total tubeless PCNLs, **b** standard versus tubeless PCNLs, and **c** tubeless versus small-bore PCNLs. OR, Odds ratio; CI, confidence interval; W, Weight

The transfusion rate did not exhibit significant differences between any of the procedures during both pairwise analysis (Fig. 12) and network meta-analysis (Fig. 7e) (Table 3). Rank probabilities demonstrated that small-bore and tubeless PCNLs with stent may be superior to the other procedures (Fig. 8e).

**Table 3:** Results of network and pairwise meta-analyses comparing procedures for transfusion rate

Procedures	Network meta-analysis		Pairwise meta-analysis^a^	
	OR	95% CI	OR	95% CI
Standard				
Total tubeless	1.27	0.35–4.40	1.17	0.41–3.30
Tubeless	2.94	0.73–14.06	3.79	0.75–19.20
Small-bore tube	2.76	0.46–24.52	NA	
Total tubeless				
Tubeless	2.37	0.34–18.19	NA	
Small-bore tube	2.39	0.24–22.21	NA	
Tubeless				
Small-bore tube	1.00	0.19–5.30	1.23	0.34–4.53

*CI* confidence interval, *OR* odds ratio

^a^Fixed-effect model with Mantel-Haenszel method

## Discussion

Conventionally, the placement of a nephrostomy tube after PCNL was considered a necessary safety option. However, the use of a nephrostomy tube has been associated with a prolonged hospital stay and more postoperative pain [18]. In 1997, Bellman et al. first reported the use of tubeless PCNL using a double-J ureteral stent and Council catheter [19]. They demonstrated that hospital length of stay, analgesia requirements, time to return to normal activities, and cost were significantly less with this procedure. Although the procedure gained popularity, tubeless PCNL with stent had two important problems: ureteral stent discomfort and loss of the advantages of a nephrostomy tube. Thus, some urologists used the approach of placing the smallest possible nephrostomy tube to minimize patient discomfort while maintaining access to the renal collecting system [20]. With the recent development of a high-density telescope, high-quality lithotripters, and radiological interventional techniques to embolize blood vessels, several investigators reported that tubeless and total tubeless (stentless) PCNL in selected patients was safe and associated with a reduced hospital length of stay and analgesic requirements.

The results of RCTs for each PCNL procedure have been reported, and previous systematic reviews and meta-analyses have been published. However, most of the studies reported in the previous meta-analyses compared standard PCNL versus tubeless PCNL with stent or standard PCNL versus total tubeless PCNL [21–25]. Therefore, an integrated analysis of standard, small-bore tube, tubeless with stent, and total tubeless PCNLs has not yet been published.

In our study, using network meta-analysis, there were no significant differences in operation time for the four procedures. It is known that large stones increase operation time and complication rates [26, 27], and operation times vary depending on the size and characteristics of the stone.

We also detected no statistically significant differences between methods for the VAS pain scores. No significant differences were observed between standard versus total tubeless PCNLs and tubeless versus small-bore tube PCNLs not only during the network meta-analysis, but even during pairwise meta-analyses. Operation-related factors that may prolong pain after PCNL include the nephrostomy tube size [28] and stent discomfort caused by a double-J stent [29], but statistically significant differences between procedures were not observed. This finding is presumably due to the relatively small sample size (only eight studies reported the VAS pain scores), and the possibility of publication bias, as suggested by the asymmetric funnel plot (Fig. 5b). However, in the rank probability test of pain scores using Bayesian Markov Chain Monte Carlo modeling, small-bore tube PCNL was ranked highest, followed by the total tubeless PCNL and then tubeless PCNL with stent (Fig. 8b). Additional RCTs are necessary in the future to more definitively address this issue.

With regard to the hemoglobin changes, network meta-analysis showed that total tubeless and small-bore tube PCNLs were superior, and tubeless with stent PCNL was the worst. In addition, total tubeless and small-bore PCNLs showed similar superiority in the network meta-analysis and rank probability test (Fig. 8c). Considering that all enrolled studies were RCTs, the possibility of selection bias between patients who had total tubeless or small-bore tube PCNLs and other procedures should be relatively low. For tubeless PCNLs, the possibility of bleeding caused by ureteral stenting should be considered. In previous studies, hematuria accounted for 13.6% of early complications and 18.1% of late complications after tubeless PCNL with stent [29]. In contrast to the hemoglobin changes, transfusion rates were not different between the four procedures. This lack of difference is likely due to the development of high-quality surgical skills and patient monitoring approaches because of the popularity of PCNL procedures.

For the length of hospital stay, the total tubeless and tubeless PCNLs showed superiority. We assumed that this is because these methods do not require additional procedures, such as nephrostomy tube removal or tract revision.

During the rank probability for each variable, small-bore and tubeless PCNLs were ranked higher for operation time, VAS pain scores, and hemoglobin change. In addition, total tubeless PCNL was ranked highest for hospital stay and transfusion rate. Notably, total tubeless PCNL was ranked highest for each item. However, total tubeless PCNL has not been in widespread use, even considering the potential benefits of this approach, because of concerns that potentially fatal complications, such as massive bleeding without a nephrostomy tube in place, may occur [30]. Because omitting a nephrostomy catheter may potentially increase the risk of bleeding and serious complications, various methods have been used in an attempt to seal the tract. Milkahi and colleagues were the first to describe the instillation of the hemostatic agent Tiseel® into the nephrostomy tract [31]. However, they were unable to determine whether this diminished postoperative bleeding or urinary extravasation following tubeless PCNL. Choi et al. instilled gel matrix thrombin (Floseal®) into the tract whenever persistent bleeding was observed after omitting the nephrostomy catheter [32]. Okeke et al. explored cryoablation of the nephrostomy tract after tubeless PCNL, where they inserted a cryoprobe into the access tract and performed a 10-min freeze-thaw cycle at a temperature -20 °C. This method did not significantly affect the rate of delayed bleeding or urinary extravasation [33]. Recently, a randomized study by Cormio et al. showed that TachoSil® provided better tract control and a shorter hospital stay than nephrostomy tube placement, although it did not reduce pain or analgesic requirements [34].

Total tubeless PCNL is advocated by leading surgeons in the field of endourology. The future role of tubed PCNL will likely reside primarily in cases of severe intraoperative bleeding or major damage to the collecting system, and when there is the possibility of a second-look operation. However, some controversies remain about the feasibility and efficacy of tubeless PCNLs in certain clinical settings. In their prospective randomized study, Shoma et al. suggested that the tubeless approach might not be suitable for patients with chronic kidney disease or those who require a supracostal approach [30]. However, Shah et al. reported the successful use of a tubeless technique in a patient with chronic kidney disease. Likewise, Sofikerim et al. reported that tubeless PCNL is a safe and effective technique, even for supracostal access, and is associated with less postoperative pain and shorter hospital stay [35]. Resorlu et al. maintained that single or no nephrostomy drainage following multitract PCNL offered the potential advantages of decreased postoperative analgesic requirements and shorter hospital stay, without increasing the rate of complications [36].

A limitation of our study was that we did not perform subgroup analyses based on the size of the stone. We also did not compare success rates because the success rates were high in each study. In addition, there was some degree of publication bias. However, in the review of 48 articles from the Cochrane Database of Systematic Reviews performed by Sutton et al., publication or related biases were noted to be common within the sample of assessed meta-analyses, but did not affect the conclusions in most cases [37]. Additionally, the position of the patient during PCNL (prone or supine position) can influence the outcomes of a tubeless or not tubeless procedure. Anesthesiologists prefer the supine position because of better airway control during procedures. Another advantage of the supine position is that there is no need for position changes when performing additional endoscopic procedures, such as cystoscopic or ureteroscopic operations [38]. Endoscopic combined intrarenal surgery is also a novel way of performing PCNL in the supine position [39]. Better visualization with the procedure allows for correct puncture of the kidney, and thus, can improve the safety and feasibility of a tubeless or total tubeless procedure.

Despite these limitations and shortcomings, our study has the substantial advantage of including larger samples from each study than the previously conducted pairwise meta-analyses [40]. Moreover, this is the first study to use network meta-analysis to compare PCNL methods, which enhances the statistical confidence and overcomes the limitations of pairwise meta-analyses.

## Conclusions

In comparing each procedure through network meta-analysis, total tubeless and small-bore PCNLs were superior in terms of hemoglobin change, and total tubeless and tubeless PCNLs were superior with regard to the length of hospital stay. These findings indicate that conventional PCNL can be replaced with other techniques, especially total tubeless PCNL, in selected patients.

## Abbreviations

PCNL: Percutaneous nephrolithotomy
RCT: Randomized controlled trial
VAS: Visual analogue scale
